# Second-line treatment strategy for urothelial cancer patients who progress or are unfit for cisplatin therapy: a network meta-analysis

**DOI:** 10.1186/s12894-019-0560-7

**Published:** 2019-12-02

**Authors:** Huitao Wang, Jianhe Liu, Kewei Fang, Changxing Ke, Yongming Jiang, Guang Wang, Tongxin Yang, Tao Chen, Xin Shi

**Affiliations:** Department of Urology, The Second Affiliated Hospital of Kunming Medical University, No.374, Dianmian Avenue, Yunnan, 650101 People’s Republic of China

**Keywords:** Urothelial carcinoma, Cisplatin-ineligible, Second-line, Meta-analysis

## Abstract

**Background:**

Second-line treatment for urothelial carcinoma (UC) patients is used if progression or failure after platinum-based chemotherapy occurs or if patients are cisplatin-unfit. However, there is still no widely accepted treatment strategy. We aimed to analyze the effectiveness and safety of second-line treatment strategies for UC patients.

**Methods:**

The PubMed, Embase, and Cochrane databases were searched for randomized controlled trials (RCTs) that included UC patients who were cisplatin-ineligible or unfit up to April 19, 2019. The primary outcomes were progression-free survival (PFS), overall survival (OS), and objective response rate (ORR).

**Results:**

Thirteen trials that assessed 3502 UC patients were included. This study divided the network comparisons into three parts. The first part contained studies comparing taxanes and other interventions; the second part assessed investigator’s choice chemotherapy (ICC)-related comparisons; and the third part assessed best support care (BSC). In the OS results of the first part, pembrolizumab (87.5%), ramucirumab plus docetaxel (74.6%), and atezolizumab (71.1%) had a relative advantage. Pembrolizumab also had advantages in ORR and severe adverse effect (SAE) results. Vinflunine and ramucirumab plus docetaxel had a relatively high surface under the cumulative ranking curve (SUCRA) rank by exploratory cluster analysis.

**Conclusions:**

This study concluded that atezolizumab and pembrolizumab are superior to other treatments, mainly in OS results, but no treatment confers a significant advantage in PFS. Pembrolizumab still has relative advantages in ORR and SAE results compared to ICC. Due to limitations, more studies are necessary to confirm the conclusions.

## Introduction

Urothelial carcinoma (UC) is the most common cancer of the bladder and upper urinary tract and is invasive and lethal, especially in advanced and metastatic patients [1, 2]. Advanced UC (AUC) patients generally have a poor prognosis, and only a few patients survive more than 5 years [3]. Platinum-based first-line chemotherapy is a standard treatment for muscle-invasive UC (MIUC) and AUC patients and mainly includes gemcitabine plus cisplatin (GC) and methotrexate, vinblastine, doxorubicin, and cisplatin (MVAC) regimens [4, 5].

Although many patients have an initial objective response when they first receive platinum-based chemotherapy, some patients still have disease progression. For those patients, second-line treatment is then used [6, 7]. There is also a group of patients who are unfit for cisplatin treatment; the most common cause is renal insufficiency or neurological disease [8, 9]. For those patients, carboplatin-based treatment is usually applied. There are many types of second-line treatment for cisplatin-progression or unfit UC patients at present, including vinflunine, taxanes, checkpoint inhibitors, antiangiogenic therapy, etc. However, there is still no widely accepted treatment strategy.

Previous meta-analyses showed that immunotherapy can significantly improve UC patients’ overall survival (OS) compared with chemotherapy, while antiangiogenic drugs combined with chemotherapy did not significantly improve patients’ progression-free survival (PFS) and OS compared to chemotherapy alone. Taxanes and vinflunine treatment had similar effects but different side effects [10, 11]. A meta-analysis analyzed the efficacy of single and double chemotherapeutic drugs as second-line treatments. Although dual-drug combinations can improve the objective response rate (ORR) and PFS, they had no obvious benefit for OS [12]. This study will analyze the safety and effectiveness of second-line treatment strategies for UC patients with cisplatin progression or who are unfit for cisplatin by network meta-analysis to provide guidance for clinical treatment.

## Methods

This network meta-analysis was reported in line with the preferred reporting items for systematic reviews and meta-analyses (PRISMA) and the PRISMA extension statement for network meta-analysis [13].

### Data sources and searches

To identify studies for inclusion in this review, we searched the following public databases: PubMed, Embase, and the Cochrane Central Register of Controlled Trials up to April 19, 2019. The search terms used in the search strategy included ((((bladder OR intravesical OR urothelial OR urethral OR urothelium OR urinary)) AND (neoplasms OR cancer OR malignant OR carcinoma OR tumor)) AND (random* OR randomized OR randomised)) AND (cisplatin OR platin OR platinum OR carboplatin). Only English-language articles were considered. The search strategy is detailed in the supplementary materials (Additional file 4: Table S1). The references of related reviews were also checked to avoid omissions.

### Study selection

Two authors independently selected the literature. In the case of any disagreements, a third author was introduced for discussion to reach a consensus. The inclusion criteria are as follows: 1, randomized controlled trials (RCTs); 2, studies that included UC patients who were cisplatin-resistant or unfit for cisplatin; 3, comparative studies on cytotoxicity therapy or immunotherapy; and 4, the outcome evaluation included one of the following: PFS, OS, and ORR. The exclusion criteria were as follows: 1, studies that compared the different doses or usage of the same drugs; and 2, studies that did not report one of the main outcomes (PFS, OS, or ORR). Conference abstracts, posters, and presentations of ongoing RCTs were also excluded because these brief reports do not contain detailed safety data.

### Data extraction

Data extraction was performed at the study level but not at the individual level. The extraction contents included the following: author name, publication year, research location, sample size, age of patients, stage of disease, progression or unfit status after cisplatin, intervention treatment, control, outcome report, and follow-up period. The primary results included PFS, OS, and ORR results. The secondary results included severe adverse effects (SAEs) according to the National Cancer Institute (NCI) Common Terminology Criteria> = grade 3. The Cochrane Collaboration method, which includes seven specified domains, was used to assess the methodological quality of the included trials [14].

### Data synthesis and statistical analysis

We used a frequentist framework, random-effects model for mixed multiple treatment comparisons. Network plots were produced for interventions and comparisons, and the node in each plot indicates the intervention regimen, while the line indicates each direct comparison [15]. We used odd ratios (ORs) and their 95% confidence intervals (Cls) to quantify the frequency of events results in the network meta-analysis, such as ORR and SAE results. Hazard ratios (HRs) and their 95% Cls were used to quantify the PFS and OS Cox regression results. The global inconsistency of the network analysis was evaluated by the fitness of the consistency model and the inconsistency model. Local inconsistency was assessed by the closed loops in the network for differences between direct and indirect comparisons. The surface under the cumulative ranking curve (SUCRA) probabilities were used to rank the treatments for each outcome [16]. Comparison-adjusted funnel plots were used to determine small-study effects in the analysis [17]. The SUCRA scores of all results were clustered by the average linkage clustering method to avoid the interference of individual deviation results on the overall results. A traditional meta-analysis was used to compare the interventions that could not be assessed by network meta-analysis. Data analyses were performed using STATA software (version 14.0; STATA Corporation, College Station, TX, USA).

## Results

### Literature search

**Fig. 1:**
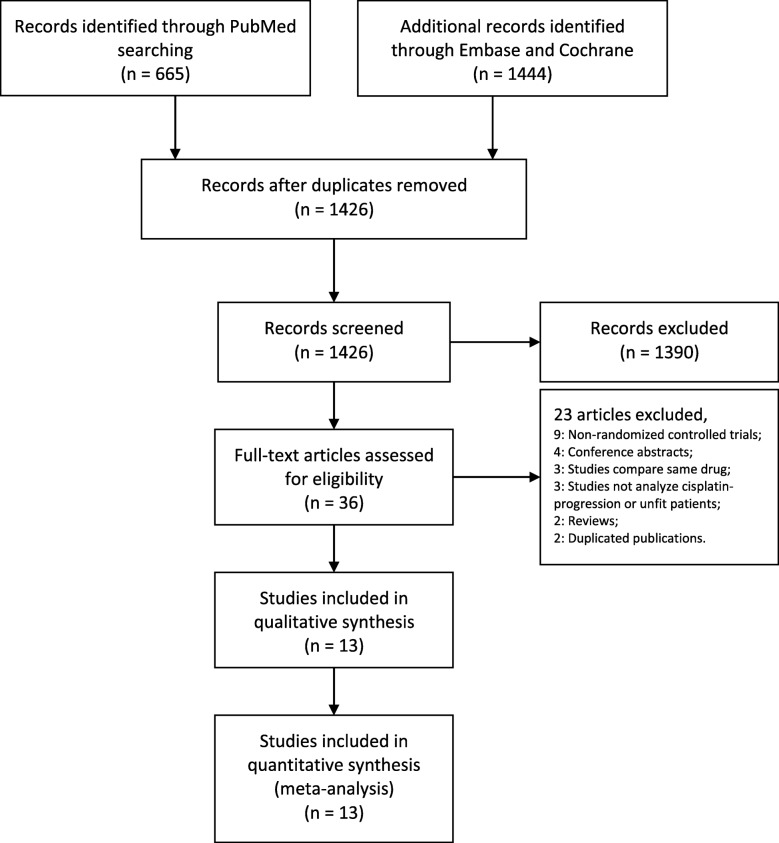
Flow diagram of the study selection process

The database searches yielded 1426 potentially relevant studies after the removal of duplications. A total of 1390 articles were excluded after screening the titles and abstracts. The full texts of the remaining 36 articles were assessed. Studies were excluded due to the following reasons: non-RCTs (9); conference abstracts (4); studies comparing the same intervention drugs (3); studies not analyzing cisplatin-progression or unfit patients (3); reviews (2); and duplicated publications (2) (Fig. 1). Finally, thirteen articles were included for further analysis [18–30].

**Fig. 2:**
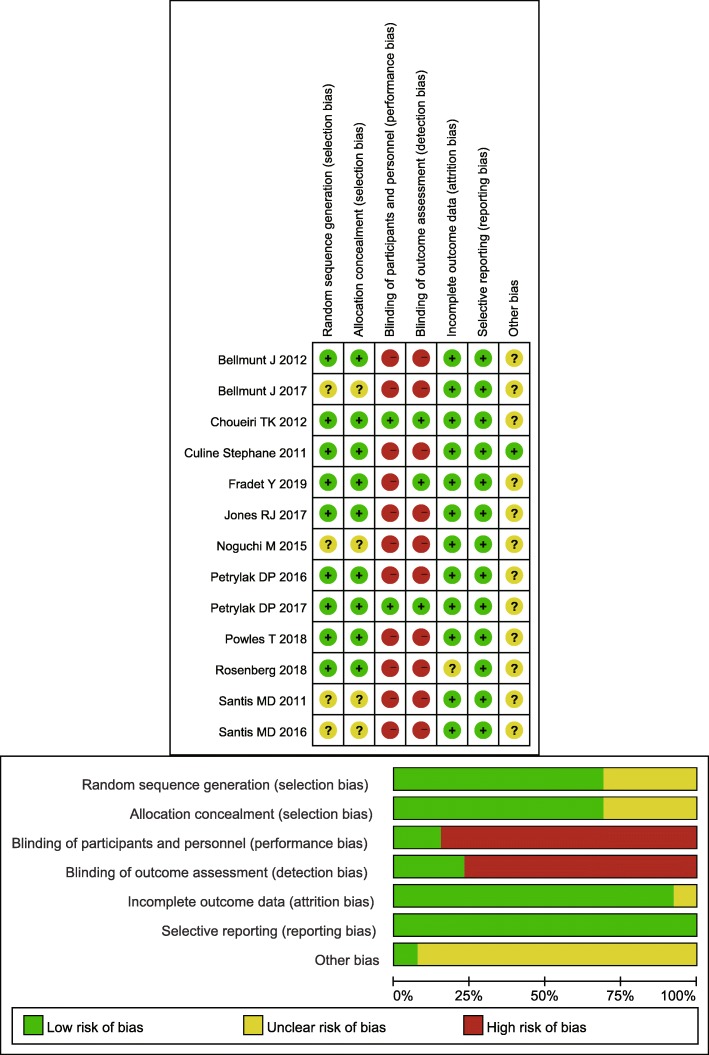
Risk of bias of the included studies

In the included studies, 3502 cisplatin-progression or unfit UC patients were analyzed. The studies were all published after 2011. Two studies were located in the UK and France, while others were multicenter studies [22, 30]. All studies included AUC or metastatic UC patients. Two studies reported the results of the same population at different follow-up periods [18, 31], and this study combined the above two results (Table 1). All the studies were performed with a randomization design. Three studies were performed with a blindness design to reduce the impact of subjective awareness on the outcomes. Overall, the quality of the included studies was ideal (Fig. 2).

**Table 1:** Characteristic of included studies

Author	Year	Location	Sample size	Title of research	Age^a^	Stage of disease	Reason of second-line therapy	Intervention	Control	Outcome	Follow-up^b^
Fradet Y [18] supplementa	2019	Multicentre	542	KEYNOTE-045	65(26–86)	Advanced	Progression	Pembrolizumab	ICC	OS;PFS;ORR;AE	Open
Rosenberg [19]	2018	Multicentre	200	Borealis-2	67(35–92)	Metastatic	Progression	Docetaxel plus Apatorsen	Docetaxel	OS;PFS;ORR;AE	Open
Powles T [20]	2018	Multicentre	931	IMvigor211	67(31–88)	Advanced or metastatic	Progression	Atezolizumab	ICC	OS;PFS;ORR;AE	Open
Petrylak DP [21]	2017	Multicentre	530	RANGE	66(32–86)	Advanced or metastatic	Progression	Docetaxel plus ramucirumab	Docetaxel placebo	PFS;ORR;AE	Open
Jones RJ [22]	2017	UK	131	NA	69(61–77)	Advanced or metastatic	Progression	Pazopanib	Paclitaxel	OS;PFS;AE;QOLS	Open
Bellmunt J [23]	2017	Multicentre	70	SECAVIN	63(35–80)	Advanced	Failure	Cabazitaxel	Vinflunine	ORR;PFS;OS	Open
Petrylak DP [24]	2016	Multicentre	148	NA	66(29–85)	Advanced or metastatic	Progression	Docetaxel plus ramucirumab	Docetaxel plus icrucumab	PFS;OS;ORR;SAE	Open
Santis MD [25]	2016	Multicentre	69	JASINT1	72(42–79)	Advanced	Unfit	Vinflunine;Gemcitabine	Vinflunine;Carboplatin	PFS;OS;SAE;ORR	25.9 M(24.3–26.5)
Noguchi M [26]	2015	Multicentre	80	NA	65(46–84)	Advanced	Progression	PPV plus BSC	BSC	PFS;OS;SAE	36 M
Bellmunt J [27]	2012	Multicentre	370	NA	NA	Advanced	Failure	Vinflunine;BSC	BSC	OS;PFS;ORR;SAE	21.5 M(16.7–25.3)
Choueiri TK [28]	2012	Multicentre	149	NA	NA	Advanced or metastatic	Progression	Docetaxel;vandetanib	Docetaxel;placebo	PFS;OS;SAE;ORR	Open
Santis MD [29]	2011	Multicentre	238	EORTC Study 30,986	71(34–87)	Advanced	Unfit	Gemcitabine;carboplatin	Methotrexate;carboplatin;vinblastine	OS;PFS;ORR;SAE	Open
Culine Stephane [30]	2011	Frence	44	GETUG V01	76(48–86)	Advanced	Unfit	Gemcitabine	Gemcitabine;oxaliplatin	ORR;OS;SAE	Open

*BSC* Best support care, *ICC* investigator’s choice chemotherapy, *NA* not available *PPV* personalized peptide vaccination

^a^Median (minimum-maximum)

^b^Open: follow-up until disease progress or patient death; M: months

This study divided network comparisons into three parts. The first part contained studies comparing taxanes and other interventions, and the interventions included apatorsen plus docetaxel, icrucumab plus docetaxel, pazopanib, ramucirumab plus docetaxel, taxane, vandetanib plus docetaxel, and vinflunine. The second part contained studies comparing investigator’s choice chemotherapy (ICC) and others, and interventions included atezolizumab, ICC, and pembrolizumab. The last part contained studies comparing best support care (BSC) and others, including personalized peptide vaccination (PPV) plus BSC, BSC, and vinflunine plus BSC. In the OS results, two ICC-related articles reported subgroup results according to different chemotherapy regimens [18, 20], so pembrolizumab and atezolizumab were also included in the OS results of the first part of the network analysis.

**Fig. 3:**
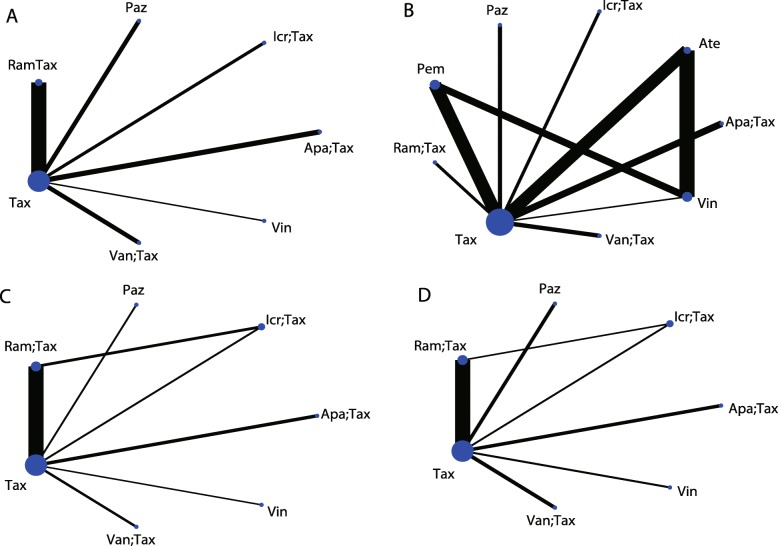
First part: network comparisons of the interventions included in the analysis. A: PFS; B: OS; C: ORR; D: SAE. Abbreviations: Apa: apatorsen; Ate: atezolizumab; Icr: icrucumab; Paz: pazopanib; Pem: pembrolizumab; Ram: ramucirumab; Tax: taxane

**Fig. 4:**
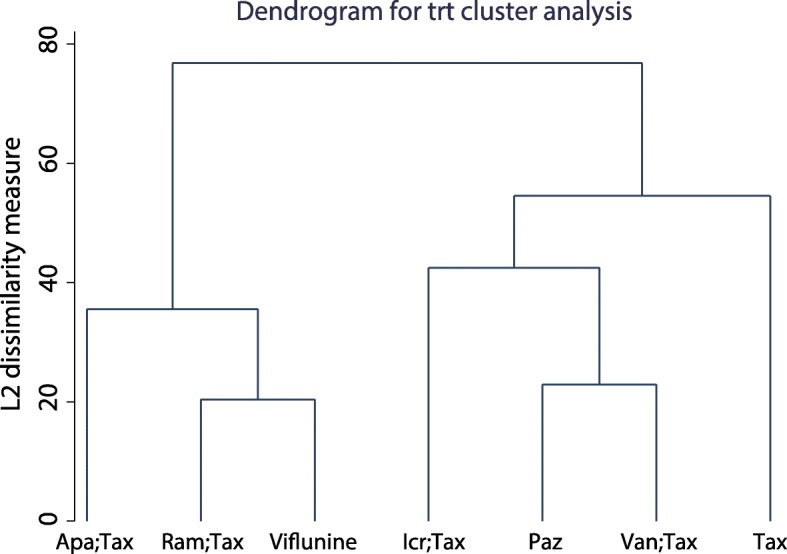
Cluster analysis of the first part of the network analysis according to the SUCRA score. Abbreviations: Apa: apatorsen; Icr: icrucumab; Paz: pazopanib; Ram: ramucirumab; Tax: taxane

**Fig. 5:**
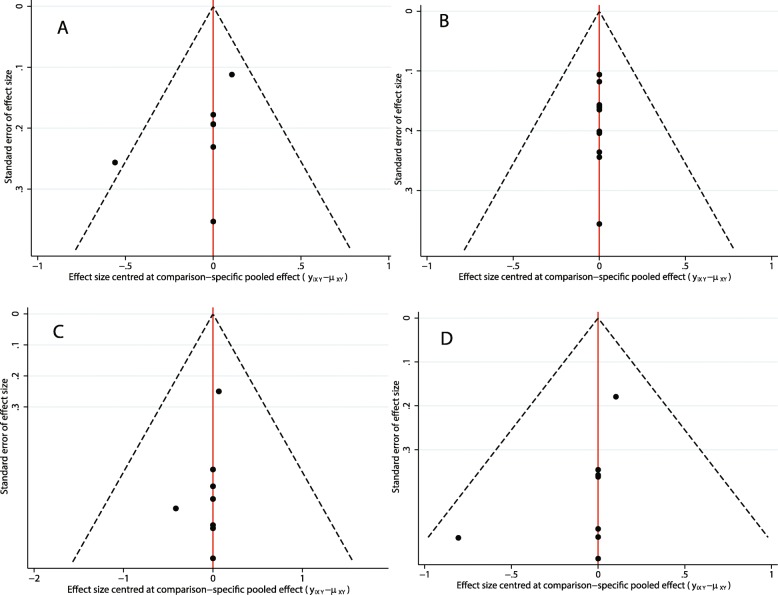
Comparisons: adjusted funnel plots of the first part of the network meta-analysis. A: PFS; B: OS; C: ORR; D: SAE

The first part of the taxane-related network analysis included PFS, OS, ORR and SAE results. Among the PFS results, there were six comparisons on taxane, among which the comparison of ramucirumab plus docetaxel and taxane was the most accurate (Fig. 3a). However, there were no significant differences among the network comparisons**.** Vinflunine and ramucirumab plus docetaxel ranked higher in the SUCRA results (Table 2). In the OS results, there were eight comparisons on taxane, three on vinflunine, two on pembrolizumab and two on atezolizumab (Fig. 3b). In the consistency analysis, no local (Additional file 1: Figure S1) and global inconsistencies (*p* = 0.2732) were found. In network comparisons, atezolizumab was found to confer a significantly longer OS than pazopanib (ln HR: 0.49; 95% CI: 0.03, 0.95) and taxane (ln HR: 0.24; 95% CI: 0.03, 0.46). Pembrolizumab was significantly superior to pazopanib (ln HR:-0.61; 95% CI: − 1.07, − 0.15), taxane (ln HR: 0.36; 95% CI: 0.15, 0.57), and vandetanib plus taxane (ln HR: 0.55; 95% CI: 0.10, 1.01). Pembrolizumab (87.5%), ramucirumab plus docetaxel (74.6%), and atezolizumab (71.1%) had a relative advantage in the SUCRA results (Table 3). For the ORR results, there were six comparisons on taxane and a comparison between ramucirumab plus docetaxel and icrucumab plus docetaxel (Fig. 3c). There were no local (Additional file 2: Figure S2) or global inconsistencies (*p* = 0.4772). Pazopanib had less ORR events compared to ramucirumab plus docetaxel (ln OR: -1.97; 95% CI: − 3.40, − 0.54) and vinflunine (ln OR: -2.27; 95% CI: − 4.32, − 0.21). Ramucirumab plus docetaxel had more events than taxane (ln OR: 0.76; 95% CI: 0.30,1.21). According to the SUCRA ranks, vinflunine (84.4%), ramucirumab plus docetaxel (82.4%), and apatorsen plus docetaxel (64.9%) have relative advantages (Table 4). There were no local (Additional file 3: Figure S3) or global inconsistencies (*p* = 0.0878) in the SAE results, and there were no significant differences in the network comparisons (Fig. 3d). Taxane had a relatively lower SAE frequency (79.8%) (Table 5). In addition to atezolizumab and pembrolizumab, exploratory cluster analysis showed that vinflunine and ramucirumab plus docetaxel had a relatively high SUCRA rank (Fig. 4). There were no small-study effects in the first part of the network analysis (Fig. 5).

**Table 2:** The league table for PFS estimates interventions according to their relative effects in first part network analysis

Apatorsen+Taxane (50.9%)^a^						
− 0.49 (− 1.90,0.93)	Vinflunine (79%)					
0.20 (− 1.09,1.49)	0.69 (− 0.73,2.11)	Vandetanib+Taxane (36.3%)				
0.22 (−0.68,1.13)	0.71 (− 0.38,1.80)	0.02 (− 0.90,0.94)	Taxane (31.7%)			
−0.35 (−1.47,0.77)	0.14 (− 1.13,1.41)	− 0.55 (− 1.68,0.58)	−0.57 (− 1.23,0.09)	Ramucirumab+Taxane (75.8%)		
0.31 (− 0.98,1.60)	0.80 (− 0.63,2.22)	0.11 (− 1.19,1.41)	0.09 (− 0.83,1.01)	0.66 (− 0.47,1.79)	Pazopanib (30.4%)	
0.08 (− 1.24,1.39)	0.56 (− 0.88,2.01)	− 0.13 (− 1.45,1.20)	−0.15 (− 1.10,0.80)	0.42 (− 0.73,1.58)	− 0.23 (− 1.56,1.09)	Icrucumab+Taxane (45.9)

*PFS* Progression-free Survival

^a^The SUCRA probabilities are performed in brackets

**Table 3:** The league table for OS estimates interventions according to their relative effects in first part network analysis

Apatorsen+Taxane (66.6%)^a^^,b^								
− 0.02 (− 0.41,0.37)	Atezolizumab (71.1%)							
0.22 (−0.35,0.80)	0.24 (− 0.27,0.76)	Icrucumab+Taxane (36.3%)						
0.47 (−0.05,0.99)	**0.49 (0.03,0.95)**^**b**^	0.25 (−0.38,0.87)	Pazopanib (11.5%)					
−0.14 (− 0.53,0.25)	−0.12 (− 0.38,0.14)	−0.36 (− 0.88,0.15)	**− 0.61 (− 1.07,-0.15)**	Pemborlizumab (87.5%)				
− 0.09 (− 0.67,0.50)	−0.07 (− 0.60,0.46)	−0.31 (− 0.99,0.37)	−0.56 (− 1.19,0.08)	0.05 (− 0.48,0.58)	Ramucirumab+Taxane (74.6%)			
0.22 (−0.10,0.55)	**0.24 (0.03,0.46)**	−0.00 (− 0.47,0.47)	−0.25 (− 0.66,0.16)	**0.36 (0.15,0.57)**	0.31 (− 0.18,0.80)	Taxane (31.6%)		
0.41 (−0.11,0.93)	0.43 (−0.02,0.89)	0.19 (− 0.43,0.81)	−0.06 (− 0.63,0.52)	**0.55 (0.10,1.01)**	0.50 (− 0.13,1.13)	0.19 (− 0.21,0.59)	Vandetanib+Taxane (16.2%)	
0.07 (− 0.34,0.48)	0.09 (− 0.11,0.30)	−0.15 (− 0.68,0.38)	−0.40 (− 0.88,0.08)	0.21 (− 0.04,0.46)	0.16 (− 0.39,0.71)	−0.15 (− 0.40,0.10)	−0.34 (− 0.82,0.13)	Vinflunine (54.6%)

*OS* Overall Survival

^a^The SUCRA probabilities are performed in brackets

^b^Bold font means significant different

**Table 4:** The league table for ORR estimates interventions according to their relative effects in first part network analysis

Apatorsen+Taxane (64.9%)^a^						
0.37 (− 1.08,1.83)	Icrucumab+Taxane (46.8%)					
1.66 (−0.02,3.33)	1.29 (− 0.44,3.01)	Pazopanib (6%)				
−0.31 (−1.40,0.77)	− 0.69 (− 1.72,0.35)	**−1.97 (− 3.40,-0.54)**^**b**^	Ramucirumab+Taxane (82.4%)			
0.44 (− 0.54,1.43)	0.07 (−1.00,1.14)	−1.22 (− 2.57,0.14)	**0.76 (0.30,1.21)**	Taxane (41.5%)		
0.93 (−0.60,2.46)	0.55 (−1.03,2.14)	−0.73 (−2.52,1.06)	1.24 (− 0.01,2.50)	0.49 (− 0.68,1.65)	Vandetanib+Taxane (24%)	
− 0.61 (− 2.44,1.22)	−0.98 (− 2.86,0.90)	**−2.27 (−4.32,-0.21)**	−0.29 (− 1.91,1.32)	−1.05 (− 2.59,0.50)	−1.54 (− 3.47,0.40)	Vinflunine (84.4%)

*ORR* Objective response rate

^a^The SUCRA probabilities are performed in brackets

^b^Bold font means significant different

**Table 5:** The league table for SAE estimates interventions according to their relative effects in first part network analysis

Apatoren+Taxane (49.6%)#						
−0.21 (−2.02,1.61)	Icrucumab+Taxane (38.5%)					
−0.25 (−2.00,1.51)	− 0.04 (− 1.85,1.77)	Pazopanib (36.7%)				
0.18 (−1.36,1.73)	0.39 (−0.98,1.76)	0.43 (−1.11,1.97)	Ramucirumab+Taxane (59.7%)			
0.47 (−0.77,1.71)	0.68 (−0.64,2.00)	0.72 (− 0.52,1.96)	0.29 (− 0.63,1.21)	Taxane (79.8%)		
−0.50 (−2.25,1.24)	− 0.29 (− 2.10,1.51)	−0.26 (− 2.00,1.49)	−0.69 (− 2.22,0.85)	−0.98 (− 2.20,0.25)	Vandetanib+Taxane (24.7%)	
0.24 (− 1.62,2.11)	0.45 (− 1.47,2.37)	0.49 (− 1.37,2.35)	0.06 (− 1.61,1.73)	− 0.23 (− 1.62,1.16)	0.75 (− 1.11,2.60)	Vinflunine (61.1%)

*SAE* Severe adverse effect

^a^The SUCRA probabilities are performed in brackets

**Fig. 6:**
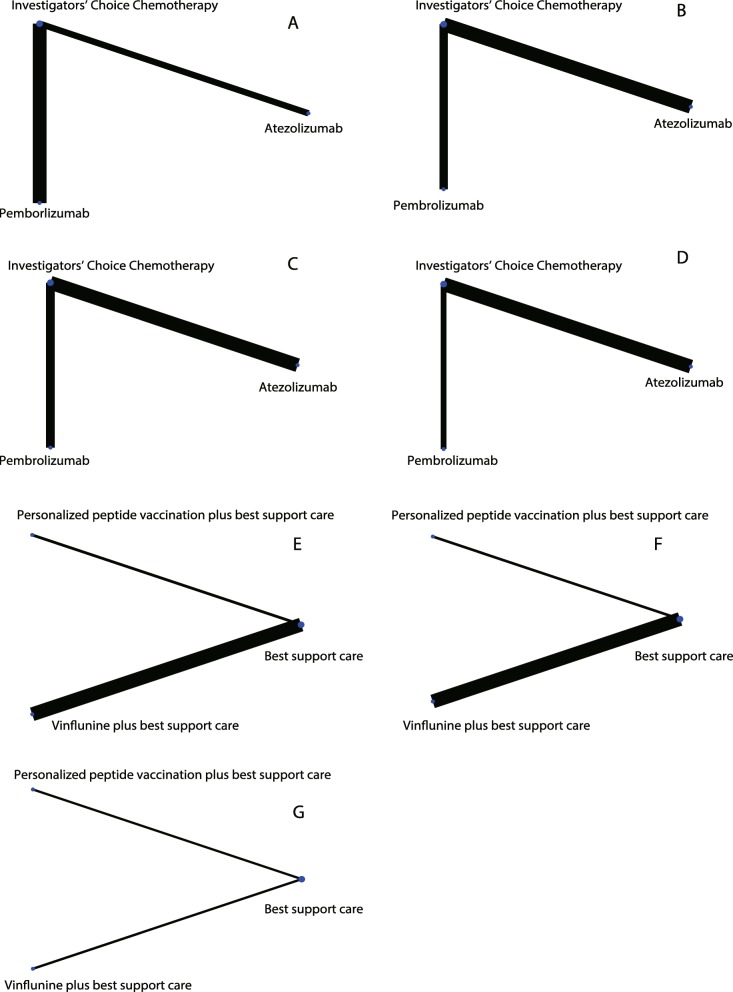
Network comparisons for interventions included in the analysis. Second part of the network: A: PFS; B: OS; C: ORR; D: SAE. Third part of the network: E: PFS; F: OS; G: ORR

The second part of the network analysis compared atezolizumab, pembrolizumab, and ICC (Fig. 6a-d). For the PFS results, there were no significant differences (Additional file 5: Table S2). For the OS results, atezolizumab (ln HR: 0.16; 95% CI: 0.01, 0.32) and pembrolizumab (ln HR: -0.36; 95% CI: − 0.55, − 0.16) were both superior to ICC (Additional file 6: Table S3). The ORR results showed that pembrolizumab is superior to atezolizumab (ln OR: -0.77; 95% CI: − 1.38, − 0.16) and ICC (ln OR: -0.77; 95% CI: − 1.25, − 0.29) (Additional file 7: Table S4). For the SAE results, pembrolizumab had a lower frequency of SAEs than atezolizumab (ln OR: 1.41; 95% CI: 0.91,1.90) and ICC (ln OR: 1.63; 95% CI: 1.21,2.04) (Additional file 8: Table S5). These results confirm the findings from the first part of the network analysis. Atezolizumab and pembrolizumab have advantages in OS. Pembrolizumab also has advantages in the ORR and SAE results. In the third part of the network analysis, BSC was evaluated as a control (Fig. 6e-g). No significant difference was found in either the PFS or OS results (Additional file 9: Table S6:Additional file 10: Table S7).

**Fig. 7:**
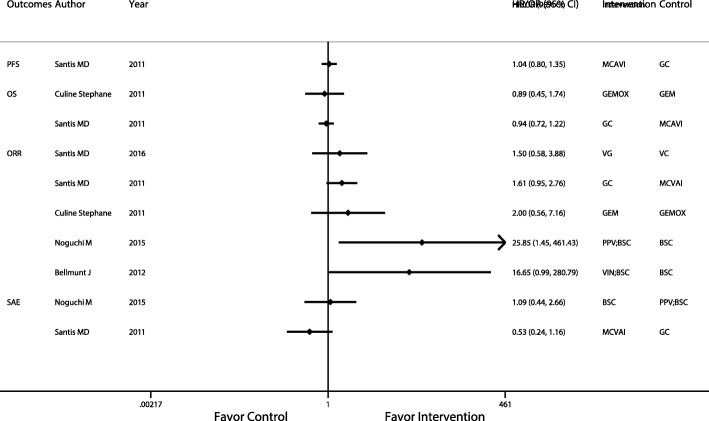
Traditional meta-analysis of comparisons that were not included in the network meta-analysis. Abbreviations: BSC: Best support care; GC: Gemcitabine plus carboplatin; GEM: Gemcitabine; GEMOX: Gemcitabine plus oxaliplatin; MCAVI: Methotrexate plus carboplatin plus vinblastine; PPV: Personalized peptide vaccination; VC: Vinflunine plus Carboplatin; VG: Vinflunine plus Gemcitabine; VIN: Vinflunine

The interventions and comparisons that did not enter the network analysis were assessed by traditional meta-analysis. In only the ORR comparison between PPV plus BSC and BSC (OR: 25.85; 95% CI: 1.45, 461.43) was there a significant difference (Fig. 7). However, there was no objective response population in the BSC group in this study, so a large standard error value reduces the accuracy of the result.

## Discussion

Platinum-based first-line chemotherapy is used to treat invasive-stage UC patients. When UC patients are cisplatin-ineligible or unfit, second-line treatment will be applied. However, the selection of second-line regimens in the clinic is still controversial. Conducting a comprehensive comparison among existing regimens and exploring more efficient and safe second-line treatments is still necessary. This work assessed the efficiency and safety of second-line therapy regimens for UC patients by network meta-analysis. The results showed that atezolizumab and pembrolizumab are superior to other treatments in only OS results, but no treatment confers a significant advantage in PFS results. Nevertheless, pembrolizumab still has relative advantages in ORR and SAE results. In addition to atezolizumab and pembrolizumab, exploratory cluster analysis showed that vinflunine and ramucirumab plus docetaxel had relatively high SUCRA ranks.

In a previous meta-analysis, immunotherapy, chemotherapy and antiangiogenesis were compared and analyzed. The results showed that immunotherapy, but not vinflunine, had more obvious benefits than taxanes regardless of PD-L1 status. Chemotherapy combined with vascular endothelial growth factor (VEGF) inhibitors did not significantly improve PFS or OS outcomes compared to chemotherapy alone [10]. These findings are similar to our results. Among the network comparisons, there was no significant advantage of intervention in PFS results, and in OS results, immunotherapy had a relative advantage.

Immune checkpoint inhibitors are a research hotspot in anticancer treatment [32]. Programmed death receptor 1 (PD-1)/programmed death ligand 1 (PD-L1) is a pair of negative costimulatory factors that play a key role in the tumor immune escape mechanism. PD-L1 can be expressed in tumor microenvironment cells and can inhibit the activation of T cells through binding to PD-1, which also weakens the entire immune system of patients [33–35]. Immune checkpoint therapy prevents PD-1/PD-L1 binding in tumors and restores the cytotoxicity of T cells [36].

Atezolizumab is a monoclonal antibody that specifically binds to PD-L1 with few serious side effects [37]. Pembrolizumab is a highly selective and humanized IgG4-k homologous PD-1 monoclonal antibody that is approved by the Food and Drug Administration of the United States for first-line AUC treatment [38]. Immune checkpoint inhibitors block the PD-1/PD-L1 connection between tumor microenvironment cells and T cells and improve the patient’s immune system. Compared with cytotoxic drugs, the OS of patients is significantly improved with this method. This improvement may be achieved through the regulation of the immune system. However, there was no difference in PFS, which may indicate the indirect effects of checkpoint inhibitors.

In one included study, subgroup analysis showed that PD-L1-combined positive scores with a cutoff between 1 and 10% could be predictive indicators that high positive-score patients have a longer OS period after checkpoint inhibitor application [31]. Unfortunately, there is still no PFS subgroup analysis report on the status of PD-L1 in this study. For other cancers, such as non-small cell lung cancer (NSCLC), an expression rate of PD-L1 greater than 50% can be used as reliable evidence for the use of immunological checkpoint drugs [39]. However, the IMvigor211 trial showed that in the PD-L1 expression rate in tumor-infiltrating immune cells in more than 5% of the population, atezolizumab did not provide significant benefits in patient survival [11, 20]. It is possible that the expression of PD-L1 in tumors or immune cells plays a more important role in guiding the application of PD-1 inhibitors than that of PD-L1 inhibitors. However, this view still needs further confirmation. Nonetheless, the above research also provides a research direction for individual treatment in clinical treatment.

PPV is also an individual treatment for patients with an improved immune system [26]. Up to four of the 31 human leukocyte antigen (HLA) vaccines were selected and applied according to the HLA type and host immunity before vaccination. However, in our research, PPV plus BSC is superior to BSC in only the ORR results but not the OS and PFS results. Nevertheless, this is still a novel attempt at individualized precise immunotherapy. Applying PPVs more conveniently and the quantity production of PPVs are the main issues.

Ramucirumab is an antagonist of VEGF. A previous meta-analysis suggested that its combination with docetaxel did not improve PFS and OS [10]. In our network comparison, ramucirumab plus docetaxel still has the advantage of a high SUCRA ranking, but there was no significant difference. However, ramucirumab had a better therapeutic effect in other tumors compared with UC, such as advanced gastric or esophagogastric junction adenocarcinoma and metastatic NSCLC [40–42]. In addition, antiangiogenic agents, such as icrucumab (targets vascular endothelial growth factor receptor 1, VEGFR1), pazopanib (VEGFR), and vandetanib (VEGFR3), also did not show obvious advantages.

Antiangiogenesis therapy does not bring obvious survival benefits to UC patients, which indicates that blocking neovascularization does not affect the invasiveness of tumors. Likewise, systemic drug administration does not block the formation of neovascularization in UC, which has a relatively poor blood supply. At the superficial UC stage, intravesical perfusion can achieve a good therapeutic effect. Further research may not be limited to novel drug development but can improve effectiveness by changing the route of medication. For example, local urethral puncture injection with a cystoscope or even surface puncture injection under the guidance of radiography can efficiently reach the tumor microenvironment.

### Limitation

Several limitations still exist. First, this study was conducted at the base trial level but not at the individual level. Second, the differences in patient characteristics between groups, such as between the cisplatin-ineligible and cisplatin-resistant patients, and the use of various ORR evaluation criteria are sources of heterogeneity in this study. Third, due to the absence of a common control, the network analysis was divided into three categories. Fourth, the impacts of drug dosage and the application period were not analyzed. Fifth, ICC and BSC interventions were selected for treatment according to the patients’ condition and to investigate judgment, so heterogeneity exists. Sixth, taxanes were considered as one type drug in this study, including docetaxel, cabazitaxel, and paclitaxel. Seventh, this study will need to be updated when new drugs and clinical outcomes emerge.

## Conclusions

At present, atezolizumab and pembrolizumab are superior to other treatments in only the OS results, but no treatment confers a significant advantage in PFS. Pembrolizumab still has relative advantages in ORR and SAE results compared to ICC.

## Supplementary information

**Additional file 1: Figure S1.** Local inconsistency plot of loop-specific heterogeneity of the OS result in the first part of the network analysis.

**Additional file 2: Figure S2.** Local inconsistency plot of loop-specific heterogeneity of the ORR result in the first part of the network analysis.

**Additional file 3: Figure S3.** Local inconsistency plot of loop-specific heterogeneity of the SAE result in the first part of the network analysis.

**Additional file 4: Table S1.** The details of the search strategy in PubMed.

**Additional file 5: Table S2**. The league table for the PFS estimates of the interventions according to their relative effects in the second part of the network analysis.

**Additional file 6: Table S3.** The league table for the OS estimates of the interventions according to their relative effects in the second part of the network analysis.

**Additional file 7: Table S4.** The league table for the ORR estimates of the interventions according to their relative effects in second part network analysis.

**Additional file 8: Table S5.** The league table for the SAE estimates of the interventions according to their relative effects in the second part of the network analysis.

**Additional file 9: Table S6.** The league table for the PFS estimates of the interventions according to their relative effects in the third part of the network analysis.

**Additional file 10: Table S7.** The league table for the OS estimates of the interventions according to their relative effects in the third part of the network analysis.

## Data Availability

The datasets supporting the conclusions of this article are included within the article and its additional files.

## Abbreviations

Apa: apatorsen
Ate: atezolizumab
AUC: advanced urothelial carcinoma
BSC: best support care
Cls: confidence intervals
GC: gemcitabine plus cisplatin
GEM: gemcitabine
GEMOX: gemcitabine plus oxaliplatin
HLA: human leukocyte antigen
HRs: hazard ratios
ICC: investigator’s choice chemotherapy
Icr: icrucumab
MCAVI: methotrexate plus carboplatin plus vinblastine
MIUC: muscle-invasive urothelial carcinoma
MVAC: methotrexate, vinblastine, doxorubicin, and cisplatin
NCI: National Cancer Institute
NSCLC: non-small cell lung cancer
ORR: objective response rate
ORs: odd ratios
OS: overall survival
Paz: pazopanib
PD-1: programmed death receptor 1
PD-L1; programmed death ligand 1; Pem: pembrolizumab
PFS: progression-free survival
PPV: personalized peptide vaccination
PRISMA: preferred reporting items for systematic reviews and meta-analyses
Ram: ramucirumab
RCTs: randomized controlled trials
SAE: severe adverse effect
SUCRA: surface under the cumulative ranking curve
Tax: taxane
UC: urothelial carcinoma
VC: vinflunine plus carboplatin
VEGF: vascular endothelial growth factor
VEGFR: vascular endothelial growth factor receptor
VG: vinflunine plus gemcitabine
VIN: vinflunine
