# Supplementary figures and images for: Second-line treatment strategy for urothelial cancer patients who progress or are unfit for cisplatin therapy: a network meta-analysis
Source: BMC Urol. 2019 Dec 2;19:125. doi: 10.1186/s12894-019-0560-7 (PMC6888906; doi:10.1186/s12894-019-0560-7)

Loops

Inconsistency  
factor

95%CI

Loop-specific  
Heterogeneity( $\tau^2$ )

Pem-Taxane-Vin

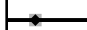

0.43

(0.00,1.23)

0.000

Ate-Taxane-Vin

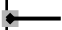

0.05

(0.00,0.82)

0.000

0 1 2

Supplement: Supplementary file 1 — Additional file 1: Figure S1. Local inconsistency plot of loop-specific heterogeneity of the OS result in the first part of the network analysis. [file 12894_2019_560_MOESM1_ESM.pdf]

Loop

Inconsistency  
Factor      95%CI

(lcr+Taxane)–(Ram+Taxane)–Taxane

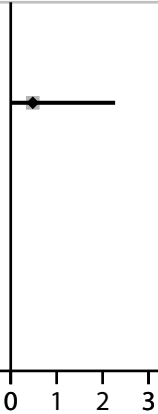

0.48      (0.00,2.27)

Supplement: Supplementary file 2 — Additional file 2: Figure S2. Local inconsistency plot of loop-specific heterogeneity of the ORR result in the first part of the network analysis. [file 12894_2019_560_MOESM2_ESM.pdf]
