# Supplementary material for: Second-line treatment strategy for urothelial cancer patients who progress or are unfit for cisplatin therapy: a network meta-analysis
Source: BMC Urol. 2019 Dec 2;19:125. doi: 10.1186/s12894-019-0560-7 (PMC6888906; doi:10.1186/s12894-019-0560-7)
Supplement: Supplementary file 3 — Additional file 3: Figure S3. Local inconsistency plot of loop-specific heterogeneity of the SAE result in the first part of the network analysis. [file 12894_2019_560_MOESM3_ESM.pdf]

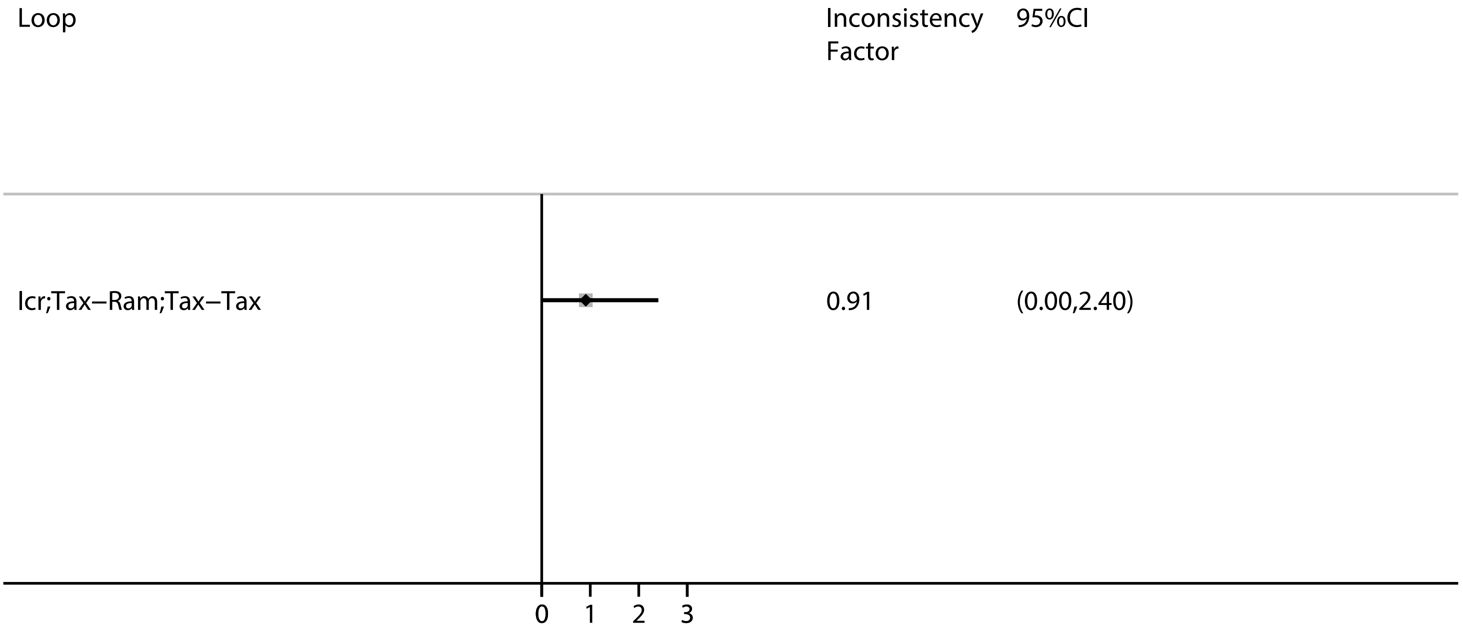

Loop
Inconsistency Factor
95%CI
Icr;Tax−Ram;Tax−Tax
0.91
(0.00,2.40)
0
1
2
3
