# Supplementary material for: Second-line treatment strategy for urothelial cancer patients who progress or are unfit for cisplatin therapy: a network meta-analysis
Source: BMC Urol. 2019 Dec 2;19:125. doi: 10.1186/s12894-019-0560-7 (PMC6888906; doi:10.1186/s12894-019-0560-7)
Supplement: Supplementary file 4 — Additional file 4: Table S1. The details of the search strategy in PubMed. [file 12894_2019_560_MOESM4_ESM.docx]

Supplementary table 1. The detail of search strategy in PubMed.

| Recent queries in pubmed | |  |
| --- | --- | --- |
| Search | Query | Items found |
| #5 | Search (((#1) AND #2) AND #3) AND #4 | 665 |
| #4 | Search (((Cisplatin) OR platin) OR platinum) OR carboplatin | 112858 |
| #3 | Search ((random*) OR randomized) OR randomised | 1284236 |
| #2 | Search ((((neoplasms) OR cancer) OR malignant) OR carcinoma) OR tumor | 4361687 |
| #1 | Search (((((bladder) OR intravesical) OR urothelial) OR urethral) OR urothelium) OR urinary | 795011 |
