# Supplementary material for: Second-line treatment strategy for urothelial cancer patients who progress or are unfit for cisplatin therapy: a network meta-analysis
Source: BMC Urol. 2019 Dec 2;19:125. doi: 10.1186/s12894-019-0560-7 (PMC6888906; doi:10.1186/s12894-019-0560-7)
Supplement: Supplementary file 5 — Additional file 5: Table S2. The league table for the PFS estimates of the interventions according to their relative effects in the second part of the network analysis. [file 12894_2019_560_MOESM5_ESM.docx]

Supplementary table 2. The league table for PFS estimates interventions according to their relative effects in second part network analysis.

| Pemborlizumab (38.9%)# |  |  |
| --- | --- | --- |
| 0.03 (-0.31,0.37) | ICC (59.4%) |  |
| -0.01(-0.30,0.28) | -0.04(-0.23,0.15) | Atezolizumab (51.7%) |

#: The SUCRA probabilities are performed in brackets.

Abbreviations: ICC: Investigator’s Choice Chemotherapy; PFS: Progression-free survival.
