# Supplementary material for: Second-line treatment strategy for urothelial cancer patients who progress or are unfit for cisplatin therapy: a network meta-analysis
Source: BMC Urol. 2019 Dec 2;19:125. doi: 10.1186/s12894-019-0560-7 (PMC6888906; doi:10.1186/s12894-019-0560-7)
Supplement: Supplementary file 6 — Additional file 6: Table S3. The league table for the OS estimates of the interventions according to their relative effects in the second part of the network analysis. [file 12894_2019_560_MOESM6_ESM.docx]

Supplementary table 3. The league table for OS estimates interventions according to their relative effects in second part network analysis.

| Atezolizumab (52.4%)# |  |  |
| --- | --- | --- |
| **0.16 (0.01,0.32)** | ICC (0.9%) |  |
| -0.19 (-0.45,0.06) | **-0.36 (-0.55,-0.16)##** | Pembrolizumab (93.4%) |

#: The SUCRA probabilities are performed in brackets.

##: Bold font means significant different.

Abbreviations: ICC: Investigator’s Choice Chemotherapy; OS: Overall survival.
