# Supplementary material for: Second-line treatment strategy for urothelial cancer patients who progress or are unfit for cisplatin therapy: a network meta-analysis
Source: BMC Urol. 2019 Dec 2;19:125. doi: 10.1186/s12894-019-0560-7 (PMC6888906; doi:10.1186/s12894-019-0560-7)
Supplement: Supplementary file 7 — Additional file 7: Table S4. The league table for the ORR estimates of the interventions according to their relative effects in second part network analysis. [file 12894_2019_560_MOESM7_ESM.docx]

Supplementary table 4. The league table for ORR estimates interventions according to their relative effects in second part network analysis.

| Atezolizumab(25.2%)# |  |  |
| --- | --- | --- |
| -0.00 (-0.38,0.38) | ICC(25.1%) |  |
| **-0.77 (-1.38,-0.16)##** | **-0.77 (-1.25,-0.29)** | Pembrolizumab(99.7%) |

#: The SUCRA probabilities are performed in brackets.

##: Bold font means significant different.

Abbreviations: ICC: Investigator’s Choice Chemotherapy; ORR: Objective response rate.
