# Supplementary material for: Second-line treatment strategy for urothelial cancer patients who progress or are unfit for cisplatin therapy: a network meta-analysis
Source: BMC Urol. 2019 Dec 2;19:125. doi: 10.1186/s12894-019-0560-7 (PMC6888906; doi:10.1186/s12894-019-0560-7)
Supplement: Supplementary file 8 — Additional file 8: Table S5. The league table for the SAE estimates of the interventions according to their relative effects in the second part of the network analysis. [file 12894_2019_560_MOESM8_ESM.docx]

Supplementary table 5. The league table for SAE estimates interventions according to their relative effects in second part network analysis.

| Atezolizumab(47.2%)# |  |  |
| --- | --- | --- |
| -0.22 (-0.49,0.05) | ICC (2.8%) |  |
| **1.41 (0.91,1.90)##** | **1.63 (1.21,2.04)** | Pembrolizumab (100%) |

#: The SUCRA probabilities are performed in brackets.

##: Bold font means significant different.

Abbreviations: ICC: Investigator’s Choice Chemotherapy; SAE: Severe adverse effect.
