# Supplementary material for: Second-line treatment strategy for urothelial cancer patients who progress or are unfit for cisplatin therapy: a network meta-analysis
Source: BMC Urol. 2019 Dec 2;19:125. doi: 10.1186/s12894-019-0560-7 (PMC6888906; doi:10.1186/s12894-019-0560-7)
Supplement: Supplementary file 9 — Additional file 9: Table S6. The league table for the PFS estimates of the interventions according to their relative effects in the third part of the network analysis. [file 12894_2019_560_MOESM9_ESM.docx]

Supplementary table 6. The league table for PFS estimates interventions according to their relative effects in third part network analysis.

| Vinflunine+BSC (53.8%)# |  | |  |
| --- | --- | --- | --- |
| -0.39 (-4.46,3.69) | | PPV+BSC (53.0%) |  |
| -0.36 (-5.84,5.12) | | 0.03(-2.34,2.40) | BSC (43.2%) |

#: The SUCRA probabilities are performed in brackets.

Abbreviations: BSC: Best support care; PFS: Progression-free survival; PPV: Personalized peptide vaccination.
