# Supplementary material for: Second-line treatment strategy for urothelial cancer patients who progress or are unfit for cisplatin therapy: a network meta-analysis
Source: BMC Urol. 2019 Dec 2;19:125. doi: 10.1186/s12894-019-0560-7 (PMC6888906; doi:10.1186/s12894-019-0560-7)
Supplement: Supplementary file 10 — Additional file 10: Table S7. The league table for the OS estimates of the interventions according to their relative effects in the third part of the network analysis. [file 12894_2019_560_MOESM10_ESM.docx]

Supplementary table 7. The league table for OS estimates interventions according to their relative effects in third part network analysis.

| BSC (34.4%)# |  |  |
| --- | --- | --- |
| -0.54 (-2.52,1.43) | PPV+BSC(67.5%) |  |
| -0.13 (-1.03,0.77) | 0.42 (-1.88,2.71) | Vinflunine+BSC (48.1%) |

#: The SUCRA probabilities are performed in brackets.

Abbreviations: BSC: Best support care; OS: Overall survival; PPV: Personalized peptide vaccination.
